# Inhalable herbal nanotherapeutics targeting lung carcinoma

**DOI:** 10.1038/s41598-025-18460-6

**Published:** 2026-02-02

**Authors:** Dina M. Gaber, Noha Nafee, Maged W. Helmy, Osama Y. Abdallah

**Affiliations:** 1 Present Address: Division of Pharmaceutical Sciences, Department of Pharmaceutics, College of Pharmacy, Arab Academy for Science, Technology and Maritime Transport, Alexandria, 1029 Egypt; 2 Department of Pharmaceutics, Faculty of Pharmacy, Alexandria University, Alexandria, 21521 Egypt; 3 Present Address: Department of Pharmaceutics, College of Pharmacy, Kuwait University, P.O. Box 24923, 13110 Safat, Kuwait; 4 Department of Pharmacology, Faculty of Pharmacy, Damanhour University, Damanhour, 22516 Beheira Egypt

**Keywords:** Solid lipid nanoparticles, Lactoferrin, Phytomedicine, Respirable microparticles, Myricetin, Phospholipid complex, Cancer therapy, Nanomedicine

## Abstract

Targeted cancer therapy promises high local drug exposure, superior efficacy and minimal hazards. Myricetin (MYR) flavonoid exhibits distinct apoptotic and antiproliferative effects on lung carcinoma. However, limited solubility and bioavailability restricted its biomedical application. Attempt for dual targeting relies on the development of receptor-mediated solid lipid nanoparticles (SLNs) and inhalation therapy. Inhalable powder was compared to non-targeted IV-administered nanocarriers. SLNs loaded with myricetin-phospholipid-complex (MYR-PH-CPX) were anchored with lactoferrin (Lf). The physicochemical properties, antitumor activity and cellular uptake were investigated. Inhalable SLN-embedded microparticles (MPs) were spray-dried, and the aerosolization parameters were determined. *In-vivo* deposition and biodistribution of targeted and non-targeted coumarin-labelled SLNs (Cou-SLNs ± Lf) were studied in mice. Lf-MYR-CPX-SLNs (< 100 nm) allowed extended MYR release > 24 h. Superior antitumor activity of Lf-MYR-CPX-SLNs was revealed by ~ 2- and 3.5-fold reduction in IC_50_ relative to MYR-CPX-SLNs and MYR-PH-CPX, respectively. Confocal imaging showed higher rate and extent of uptake of Lf-Cou-SLNs in A549-cells. MYR-SLN-embedded MPs were suited for bronchial deposition (MMAD 2.81 µm, FPF 80.5%), while Lf-Cou-SLNs-MPs ensured 1.5-fold *in-vivo* pulmonary deposition and limited migration to other body organs relative to IV-administered counterparts. The developed targeted inhalation therapy achieved efficient anti-tumor effect, targetability, preferential pulmonary deposition and low off-target biodistribution, which augmented the safety threshold.

**Supplementary Information:**

The online version contains supplementary material available at 10.1038/s41598-025-18460-6.

## Introduction

Lung cancer represents one of the most prevalent morbidity and mortality risks globally^1^. For decades, chemo-, immuno-, radiotherapy and/or surgery remain the approved treatment regimens for cancer^1^. Yet, preserving the integrity of healthy tissues while combating malignant cells represents a persistent challenge. As an alternative to harsh chemotherapeutics, phytomedicines have shown promise in treating various health conditions^2,3^. The anticancer, anti-inflammatory and anti-oxidant activities of several polyphenols and flavonoids among which myricetin (MYR), curcumin and quercetin were extensively evidenced^2,4^. In lung cancer, MYR^5^, resveratrol^6^ and berberine^7^ showed remarkable effect^4^. Their natural origin grants superior safety and minimal health hazards^3^. However, the restricted aqueous solubility and instability triggered inadequate permeability and poor pharmacokinetics; a bioavailability < 10% was recorded for myricetin and curcumin^8^.

Herbal nanomedicine recently ameliorated the effectiveness of herbal therapy. The application of nanotechnology in the formulation and delivery of herbal drugs showed significant progress^7,9^. The tiny size of nanocarriers provided unique surface, structural, electrical, mechanical and magnetic characters that could overcome the delivery challenges of phytomedicines and provide improved solubility, high payload, effective protection, controlled release and superior uptake. On this basis, thousands of scientific research studies developed nanoformulations (e.g., polymeric nanoparticles, nanocapsules, phytosomes, liposomes, lipid-based nanocarriers and liquid crystals) encapsulating various herbal drugs^5,7,8,10^.

Recent advances in cancer therapy rely on targeted therapy of herbal anticancer drugs to reduce detrimental side effects of chemotherapeutics and subsequent damage of healthy cells^1^. Despite the ability of nanoparticles for passive targeting via enhanced permeability and retention, active ligand targeting provides superior level of selectivity. Moreover, inhalation therapy grants local delivery to the lungs relative to the systemic route. In previous studies, our group prepared MYR-phospholipid complex (MYR-PH-CPX), which was then encapsulated in solid lipid nanoparticles (MYR-CPX-SLNs)^11^. MYR-CPX-SLNs reduced the IC_50_ from 180 to 35 µM following overnight incubation with A549 cell line^5^. Herein, further specificity and safety were accomplished by anchoring lactoferrin (Lf) glycoprotein to SLN surface. Selective binding of Lf to receptors expressed on the apical surface of bronchial epithelial cells (BEAS-2B) assures active targeting of lung cancer^12,13^.

Lactoferrin is an iron-binding glycoprotein (80 KDa), composed of 700 amino acids held together by disulfide bonds^14^. Research indicated that bovine Lf effectively limited the development of various types of cancer in rats when given orally during the post-initiation phases^13,15^. Its anti-cancer efficacy can be achieved via different mechanisms^16–18^; Lf has innate apoptotic and antiproliferative effect on cancer cells^13^. It also promotes the regeneration of WBCs and RBCs following chemotherapy^16^. Therefore, Lf can serve as a good ligand for coupling with solid lipid nanoparticles (SLNs) to provide a promising pulmonary drug carrier^19^.

The study herein attempts to develop Lf-coupled SLNs loaded with MYR-phospholipid complex. Selective intracellular delivery of the cargo into adenocarcinoma human alveolar cells and effective antitumor activity relative to uncoupled SLNs and free MYR were addressed. For pulmonary application, a dry powder inhaler (DPI) comprising spray-dried SLN-embedded microparticles was prepared and characterized in terms of morphology, flow properties and aerodynamic behavior. The pharmacodynamics of inhaled particles were compared to IV-administered SLNs in mice.

## Results and discussion

### Lactoferrin-targeted *versus* untargeted MYR-SLNs

#### Colloidal properties and morphology of SLNs

**Fig. 1:**
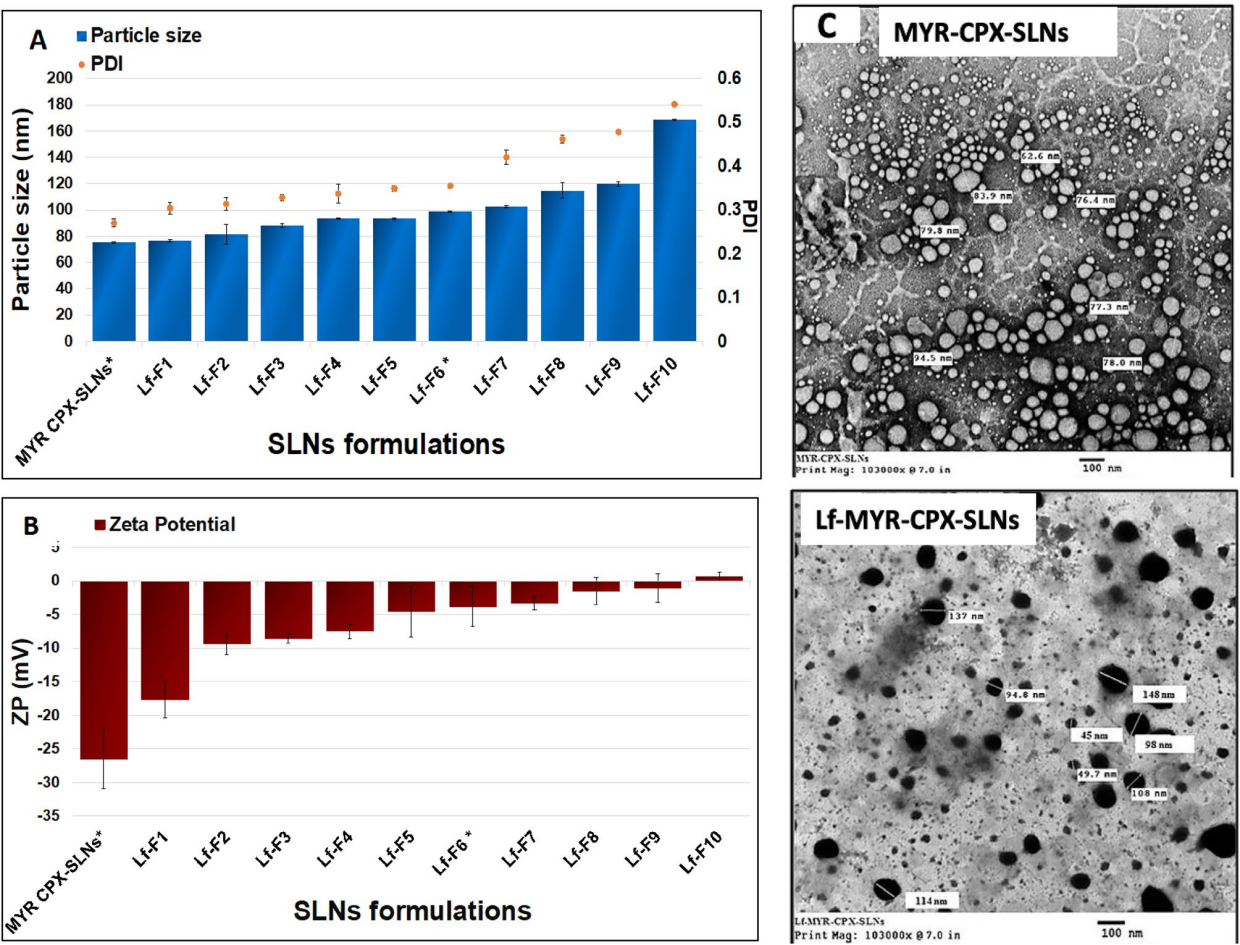
(**A**) Mean particle size and PDI of different MYR-CPX-SLNs and Lf-MYR-CPX-SLNs; (**B**) Zeta potential of different MYR-CPX-SLNs and Lf-MYR-CPX-SLNs; (**C**) TEM microphotograph of MYR-CPX-SLNs and Lf-MYR-CPX-SLNs-F6.

Ten SLN formulations (Lf-F1–Lf-F10) coupled with increasing concentrations of Lf (0.2–6% w/v) were prepared, Table S1 (Supplementary materials). As depicted from Fig. 1A, the particle diameter increased gradually from 75.28 to 168.3 nm as well as the PDI. This is analogous to previously reported results indicating that Lf-coupled rifampicin-loaded SLNs were 35 nm larger than uncoupled SLNs^15^. Meanwhile, the zeta potential reverted from negative − 17.7 ± 2.73 mV (Lf-F1 SLNs) to positive 0.6 ± 0.66 mV (Lf-F10 SLNs) by increasing Lf concentration, Fig. 1B, which partially confirms coupling of the positively-charged amine groups of Lf to SLN surface. The weak zeta potential of Lf-F10 SLNs can be correlated to the doubling in size and possible SLN agglomeration. SLNs were mainly stabilized by Gelucire 50/13 which is a water dispersible surfactant composed of glycerides and polyethylene glycol esters with an HLB value of 13 and a melting range of 50–55 °C. Being non-ionic in nature, G50/13 cannot impart high zeta potential to ensure electrostatic repulsion, however its hydrophilic characteristics can provide good coating of particle surface and sufficient steric stabilization.

Based on the colloidal characteristics, formula Lf-F6 provided uniform size distribution together with sufficient functionalization with lactoferrin. Accordingly, formula Lf-F6 SLNs was selected for further investigations and subsequently referred to as Lf-MYR-CPX-SLNs.

Labelled SLNs (Cou 6-SLNs and Lf-Cou 6-SLNs) showed an average diameter of 66.3 ± 0.91 and 81.16 ± 0.97 nm, respectively. Labelled SLNs were comparatively smaller than MYR-loaded SLNs. The effect of drug loading on particle diameter can be directly linked to the molecular weight and physicochemical properties of the cargo. Coumarin-6 with a small molecular weight (MWt, 146.143 g/mol) did not show an obvious increase in nanoparticle size. In comparison, loading SLNs with myricetin (MWt 318.235 g/mol) complexed with the phospholipid Lipoid S100 (MWt 206.33 g/mol) resulted in relatively larger SLNs (98.6 nm). In either case, anchoring Lf (1% w/v) to particle surface increased the mean diameter by 16 and 19 nm for Lf-Cou-SLNs and Lf-MYR-CPX-SLNs, respectively.

The morphology of both Lf-coupled (Lf-MYR-CPX-SLNs) and uncoupled SLNs (MYR-CPX-SLNs) showed spherical particles with smooth surface, Fig. 1C. Interestingly, ligand-coupled SLNs appeared darker, consistent with the TEM photomicrographs reported by Shilpi et al*.*^15^.

#### Qualitative and quantitative confirmation of Lactoferrin coupling to MYR-SLNs

**Fig. 2:**
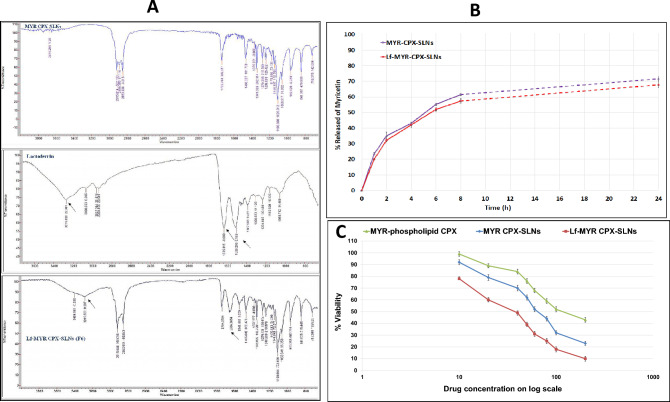
(**A**) FTIR spectra of MYR-CPX-SLNs, lactoferrin and Lf-MYR-CPX-SLNs (Lf-F6); (**B**) In vitro release of MYR from MYR-CPX-SLNs and Lf-MYR-CPX-SLNs, mean ± S.D., (n = 3); (**C**) Viability of A549 cells measured by the MTT assay following overnight exposure to various concentrations of MYR-phospholipid CPX, MYR-CPX-SLNs and Lf-MYR-CPX-SLNs at 37 ± 0.5 °C, mean ± S.D., (n = 3).

The coupling efficiency of Lf to SLNs was validated using FT-IR. The spectrum of free Lf exhibited characteristic protein band peaks, including –N–H stretching of amide I at 3288 cm⁻^1^, C = O stretching vibration related to (amide II) peptide group at 1635.815 cm⁻^1^, and N–H bending with contributions from C–N stretching vibrations at 1508.298 cm⁻^1^ (Fig. 2A).

The disappearance of the amide peak in the spectrum of Lf-MYR-CPX-SLNs confirmed the successful attachment of Lf, presumably attributed to the electrostatic interaction between the cationic targeting ligand and the anionic surface of SLNs. This is consistent with findings previously reported for bovine lactoferrin-loaded liposomes and SLNs^20^.

For quantitative estimation of coupling efficiency, Bradford assay reflected a concentration of 9.38 mg/ml Lf bound to Lf-MYR-CPX-SLNs (Lf-F6, Table S1), which represents a high coupling efficiency of 95.43%.

#### The encapsulation efficiency and in vitro release of MYR

High EE of MYR was recorded for MYR-CPX-SLNs and Lf-MYR-CPX-SLNs (97.9 ± 0.14% and 95.3 ± 0.5%, respectively). Of interest, promising encapsulation of Cou-6 in Lf-Cou-SLNs and Cou-SLNs was estimated (96 and 98.4%), respectively, highlighting the potential of these SLNs for cellular uptake and biodistribution investigations.

MYR release from coupled and uncoupled SLNs was analyzed by the dialysis method. After 24 h, 71.5% and 67.7% MYR was released from MYR-CPX-SLNs and Lf-MYR-CPX-SLNs, respectively (Fig. 2B). Lf is likely to offer considerable shielding onto NPs surface. Comparable release patterns have been observed for paclitaxel and rifampicin from both uncoupled and Lf-coupled SLNs^15,21^ as well as for methotrexate released from lactoferrin–dendrimer conjugates^22^.

### In vitro assessment on pulmonary cell culture models

#### The anti-tumor activity of MYR-SLNs by MTT assay

The cytotoxic effect of Lf-MYR-CPX-SLNs on A549 cells was explored relative to MYR-CPX-SLNs and MYR-phospholipid complex to reflect their antitumor activity.

Actively-targeted SLNs showed the lowest viability profile, Fig. 2C. At MYR concentration < 20 µM, the viability of A549 cells was between 60 and 89%. A drop in viability to 10% was observed following incubation with Lf-MYR-CPX-SLNs (equivalent to 200 µM MYR) relative to 23 and 43% for MYR-CPX-SLNs and MYR-CPX, respectively. The significant superior cytotoxicity related to lactoferrin ligand (Two-way ANOVA, p = 0.000002) is translated to 2- to fourfold reduction in IC_50_ value, Table 1.

**Table 1:** IC_50_ values for MYR formulations following 24 h incubation with A549 cells.

Formulation	IC_50_ (µM)
MYR-CPX	113.8
MYR-CPX-SLNs	67.29
Lf-MYR-CPX-SLNs	35.01

Lactoferrin (Lf) exists in three isoforms: the α isoform, which exhibits iron-binding capability, and the β and γ isoforms, which lack iron-binding properties but possess ribonuclease activity^23^. The biological functions of lactoferrin include anticancer, anti-inflammatory, antibacterial, antifungal, antiviral, and immunoregulatory activities^19,23^. We previously reported 33% reduction in IC_50_ for MYR-phospholipid complex compared to free MYR^5^. Other studies revealed an IC_50_ value of 229 µM for MYR^24^. Lactoferrin ligand targeting was also recorded to potentiate the antitumor activity of Lf- paclitaxel (PTX)-loaded SLNs on BEAS-2B, where the IC_50_ values were 7.5, 4.6 and 1.1 µg/ml for free PTX, PTX-SLNs and Lf-PTX-loaded SLNs, respectively^21^.

#### Uptake efficiency of SLNs and colocalization studies

##### Effect of the incubation time on the uptake of SLNs in A549 cells

**Fig. 3:**
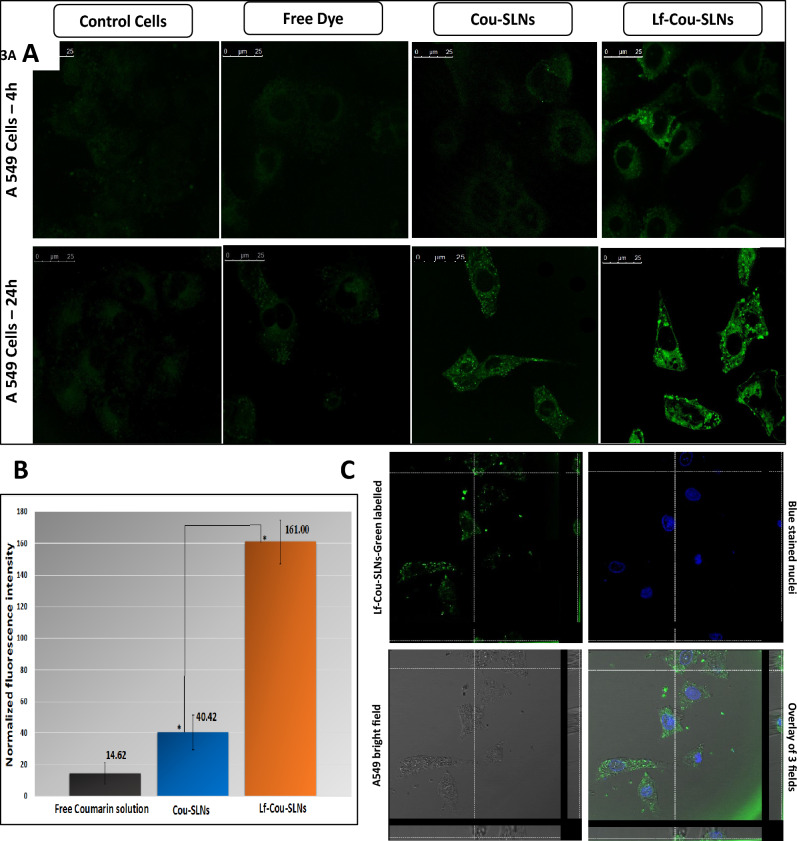
(**A**) CLSM images of A549 cells incubated with free cou solution, Cou-SLNs and Lf-Cou-SLNs for 4 and 24 h (Magnification 63X, Scale bar, 25 µm); (**B**) Normalized fluorescence intensity recovered from A549 viable cells after 24 h treatment with free dye, Cou-SLNs or Lf-Cou-SLNs. Statistically significant differences from Lf-Cou-SLNs and Cou-SLNs to untreated cells (p < 0.05); (**C**) 3D-time laps confocal images of the cellular uptake of Lf-Cou-SLNs in A549 lung cancer cells after 24 h (Z-stacks 14 µm, Scale bar, 25 µm).

The cellular uptake of Cou-SLNs, Lf-Cou-SLNs, and the free dye was evaluated in A549 cells over 4 and 24 h using CLSM. Following a 4 h incubation, Cou-SLNs and the free dye exhibited weak green fluorescence analogous to that of the control cells (Fig. 3A). In contrast, Lf-Cou-SLNs displayed distinct green fluorescence spots. Prolonged incubation with cells (24 h) resulted in enhanced uptake of all tested samples (Fig. 3A), whereby Lf-Cou-SLNs demonstrated significantly brighter fluorescence, indicating a higher degree of internalization. This observation aligns with Nafee et al.^5^ reporting 2.5-time higher fluorescence intensity of internalized Cou-SLNs than that of the free coumarin. In the current study, Lf-Cou-SLNs demonstrated the fourfold increase in fluorescence intensity in A549 cells over Cou-SLNs as estimated by Leica AF software (Fig. 3B). The significant difference (One-way ANOVA, p < 0.05) underscores the substantial impact of ligand targeting on intracellular drug delivery.

##### Colocalization of labeled SLNs in A549 cells

To differentiate between the binding of SLNs to cell surface and actual internalization, 3D time-lapse imaging was performed (Fig. 3C). Forty z-stacks (14 µm in the z-direction) confirmed that the particles were localized primarily in the cytoplasm with occasional dispersion in the blue-stained nuclei. Lf-Cou-SLNs were distinctly visible as sharp green fluorescent spots, which suggests their potential entrapment in intracellular vesicles and the critical role of active ligand targeting in enhancing cancer nanotherapeutics. In line with that, Liu et al.^25^ demonstrated that the cell-penetrating peptide (RRWQW) derived from bovine lactoferrin could effectively deliver plasmid DNA into A549 cells.

### Physicochemical characteristics of SLNs-embedded respirable microparticles

While inhalation is crucial for effective treatment of lung diseases, the pulmonary deposition of NPs is challenging owing to premature exhalation. Additionally, the strong interparticulate forces may lead to uncontrolled aggregation during administration via passive DPIs. Attempts to convert nanoparticles into micro-scale nanocomposites referred to as nano-embedded microparticles with an aerodynamic diameter between 1 and 5 μm have been reported^26–28^.

#### Particle size of SD-MPs

To grant adequate pulmonary drug deposition in the lung, MYR-CPX-loaded SLNs were spray dried with a carrier blend of mannitol, maltodextrin and L-leucine at varying proportion to produce SD-MPs. Spray drying of MYR-CPX-SLNs alone without carrier produced SD-MPs with large particle size (Dv_50_ = 22.03 ± 2.50 μm) revealing possible agglomeration and/or aggregation. As depicted from Table 2, smaller Dv_50_ (4.5–6.9 μm) and theoretical aerodynamic diameter < 5 μm were recorded for SD-MP1 to SD-MP4 revealing their convenience for pulmonary application. All formulae showed monomodal particle size distribution. The span values (1.24–1.84) further proved the narrow size distribution. SD-MP3 containing mannitol, maltodextrin and L-leucine (1.5:0.75:0.75%w/v, respectively) was selected for further investigation of the aerodynamic behavior.

**Table 2:** Particle size distribution data of spray dried MYR-microparticles.

Formula code	Dv_10_ (µm)	Dv_50_ (µm)	Dv_90_ (µm)	Theoretical calculated aerodynamic diameter (µm)	Span index
SD-MYR-CPX-SLNs	7.37	22.03	42.51	8.67	1.60
SD-MP1	1.84	6.9	10.37	4.40	1.24
SD-MP2	1.46	5.19	9.98	2.57	1.64
SD-MP3	1.72	4.5	10	2.39	1.84
SD-MP4	1.67	5.94	12.3	3.24	1.79

The matrix formers, mannitol and maltodextrin, were picked for their ability as drying protectants during water removal^29^. They also serve to shape the MPs and modulate particle/particle interactions^30^. The amino acid L-leucine enhances aerosolization and powder dispersibility by creating a shell on the particle surface upon drying, which prevents particle fusion and preserves the individual MP^29,30^.

#### Microparticle yield and drug content

Spray drying yielded 28.75 to 89.05% w/w microparticles according to the carrier composition, Table 3. The combination of maltodextrin with mannitol (SD-MP3, SD-MP4) significantly increased particle yield compared to SD-MP2 (One-way ANOVA, p < 0.05). This aligns with prior research indicating the addition of dextran to mannitol effectively prevented particle shrinkage and collapse by altering the glass transition temperature T_g_ (collapse temperature). The low T_g_ of mannitol, along with its rubbery state, predominantly contributes to adhesion to the spray dryer^29^. Similarly, the combination of low and high molecular weight sugars enhanced the spray drying yields^31^.

**Table 3:** Characteristics of spray dried MYR-microparticles (Mean ± S.D., n = 3).

	Formula code				
	SD-MYR-CPX-SLNs	SD-MP1	SD-MP2	SD-MP3	SD-MP4
Yield (%w/w)	28.75 ± 1.77	68.8 ± 1.06	80.5 ± 0.71	89.05 ± 0.64	86.15 ± 0.92
% Drug recovery	73.3 ± 1.70	90.50 ± 0.71	92.65 ± 1.91	95.15 ± 1.20	93.7 ± 0.99
Angle of repose (θ)	67.2 ± 0.71	46.7 ± 2.12	32.6 ± 0.42	29.3 ± 0.14	38.65 ± 0.85
Bulk density (g/cm^3^)	0.091 ± 0.03	0.265 ± 0.014	0.2079 ± 0.022	0.247 ± 0.053	0.2483 ± 0.007
Tapped density (g/cm^3^)	0.155 ± 0.09	0.407 ± 0.12	0.2445 ± 0.005	0.2823 ± 0.42	0.2979 ± 0.26
CI (%)	41.3 ± 0.3112	34.9 ± 0.0246	14.96 ± 0.317	12.5 ± 0.09	16.64 ± 0.075
HR	1.7 ± 0.16	1.53 ± 0.07	1.17 ± 0.092	1.143 ± 0.12	1.19 ± 0.088

The MYR content across the various formulations (SD-MP1 to SD-MP4) ranged from 90.5–95.15% relative to the theoretical content (Table 3) revealing even distribution of the active ingredient in various SD-MP formulations.

#### The flow properties

Good flow properties are considered the key factor for efficient aerosolization of respirable particles. The angle of repose θ, Hausner ratio (HR) and Carr’s index (CI) of SD-MPs were depicted in Table 3. Carrier-free spray dried MYR-CPX-SLNs formed a sticky powder exhibiting strong inter-particulate cohesion, and subsequently poor flow properties as revealed by the large angle of repose, CI and HR (67.2°, 41.3% and 1.7, respectively). The matrix formers significantly enhanced the flow properties in comparison to carrier-free spray dried SLNs. The incorporation of mannitol (SD-MP1) as a matrix former resulted in significant reduction in the flow indices, as mannitol is known for its low compressibility value. The introduction of L-leucine (SD-MP2) potentially reduced particle surface energy, water sorption and particle cohesion; a powder with good flow properties is thus obtained. Furthermore, leucine is reported to induce surface irregularities that enlarge the inter-particular spaces and thus reduce particle–particle interactions^32^. The combination of maltodextrin with mannitol in SD-MP3 in ratio 0.75:1.5 w/w showed a good flow characteristic relative to SD-MP4 containing maltodextrin: mannitol (1.5:0.75 w/w) as evidenced by the low θ, CI and HR values of 29.3°, 12.5% and 1.14, respectively, Table 3.

#### Microparticle morphology

**Fig. 4:**
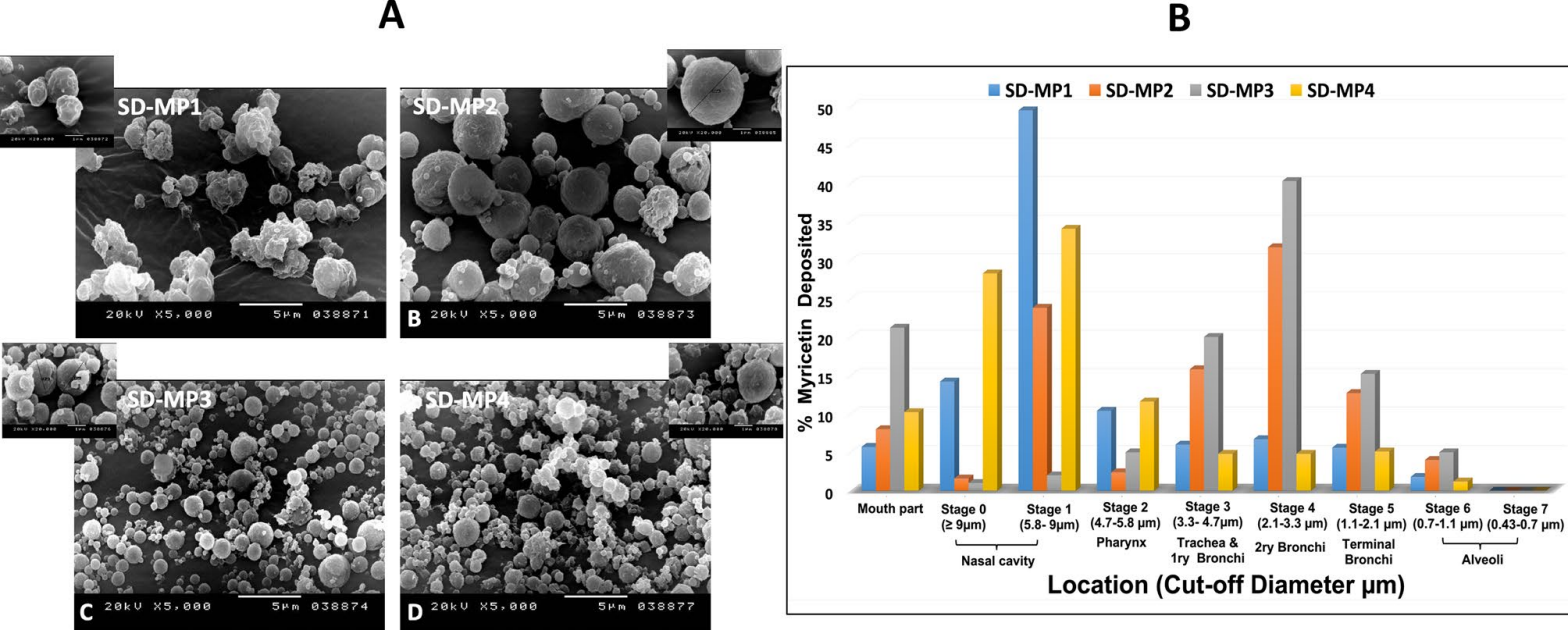
(**A**) SEM micrographs of SLN-embedded microparticles SD-MP1, SD-MP2, SD-MP3, and SD-MP4 (Magnification power 5000X); (**B**) Pulmonary deposition of SLN-embedded microparticles (SD-MP1, SD-MP2, SD-MP3, and SD-MP4) at different stages of the NGI.

SEM photomicrographs of spray-dried SLN-embedded microparticles (SD-MP1 to SD-MP4) were illustrated in Fig. 4A. MYR-CPX-SLNs spray-dried with mannitol (SD-MP1) produced distinct spherical particles with a slightly corrugated surface, while leucine-containing particles (SD-MP2) revealed enhanced surface roughness and corrugation. Due to its hydrophobic water repellent nature, leucine decreases moisture absorption and diminishes particle cohesion^29,33^. Spherical particles with less corrugated surfaces were produced by mixing maltodextrin with mannitol in both SD-MP3 and SD-MP4 (Fig. 4A). Altering mannitol-to-maltodextrin ratio consistently influenced microparticle morphology by mitigating their shrinkage and collapse, and modifying their glass transition temperature (Tg).

#### Drug-excipient compatibility in SD-MPs

##### Differential scanning calorimetry

The DSC thermograms of individual components as well as microparticle formulations (SD-MP2 and SD-MP3) were presented in Fig. S.1A-F (Supplementary material). MYR showed a single characteristic endothermic peak at 314.16 °C (Fig. S.1A). In contrast, maltodextrin displayed no endothermic peaks, confirming its amorphous nature (Fig. S.1B). Mannitol and leucine exhibited a characteristic endothermic peak corresponding to their melting point at 168.9 and 263.82 °C, respectively (Fig. S.1C-D). In the case of SD-MPs (SD-MP2/ SD-MP3), the characteristic peak of MYR could not be detected after spray drying, whereas the endothermic peaks corresponding to leucine and mannitol remained visible (Fig. S.1E-F).

##### FTIR spectroscopy

IR spectra of individual microparticle components, SD-MPs and spray-dried MYR-CPX-SLNs were illustrated in (Fig. S.2-Supplementary material). MYR has 4 characteristic peaks for IR absorption at wave number (cm^-1^) 3586.65–3282.31 (O–H stretching), 2942.33, 2855.4 (C-H stretching), 1660.5 (-C = O group), and 1611.71–1517 (-C = C). Detailed IR spectra for the individual carriers are described in the supplementary materials. FT-IR spectra of SD-MPs indicated the absence of the characteristic MYR peaks (Fig. S.2—Supplementary material), presumably related to the drug overlap by an abundance of carriers. In line with that, Ishak and Osman^29^ noted the disappearance of the distinctive peaks of atorvastatin from the IR spectra of spray-dried self-microemulsifying powders.

#### Aerodynamic parameters of SD-MPs

For competent tumor inhalation therapy, the microparticles should target the tumor site with minimal/no systemic exposure. The aerosol performance of SD-MPs was investigated by the NGI and interpreted by the aerodynamic parameters MMAD, FPF, GSD and EF (Table 4). The deposition pattern of different spray-dried microparticles was illustrated in terms of amount deposited in µg per stage and percentage in Fig. S.3 and 4B, respectively. Generally, a minimum % deposited in the upper airways would be favored. Interestingly, SD-MP3 ensured minor MYR deposition (16%) in the initial stages of the NGI (S0-S2) compared to more than 60% determined for SD-MP1, Fig. 4B. Using mannitol alone for spray drying (SD-MP1) might lead to elimination of the vast majority of the particles from the airways by mucociliary clearance. Comparatively, leucine as a surface active molecule reduced early particle deposition to ~ 40% (SD-MP2). Meanwhile, SD-MP3 showed preferential deposition in the bronchial region (~ 78% corresponding to stages 3–5), which was superior to SD-MP4 containing different mannitol: maltodextrin ratio. The potential of leucine to enhance particle dispersibility and limit their cohesiveness through the formation of a shell on the particle surface was reported. Meanwhile, decreasing the surface free energy reduces the particle tendency to agglomerate^34^. The same was observed during spray drying of DNase microparticles in presence of leucine, where the emitted fraction increased from 75 to 96% with a respirable fraction (RF) of 68%.

**Table 4:** Aerodynamic parameters of spray dried MYR-microparticles.

Formula code	MMAD (μm)	EF (%)	FPF (%)	GSD
SD-MYR-CPX-SLNs	6.64	77.5	20.1	1.34
SD-MP1	3.2	91.2	64.2	1.59
SD-MP2	2.77	93	81.23	1.48
SD-MP3	4.68	82	45.12	1.82
SD-MP4	3.38	86.6	53.7	2.54

SD-MP2 demonstrated the most favorable aerosolization profile (Table 4), consistent with a previously reported formulation characterized by MMAD of 2.77 μm, FPF of 81.23%, and EF of 93%^5^.

For the in vivo biodistribution studies, coumarin-labeled microparticles (SD-Cou MP) analogous to formula SD-MP2 were prepared by substituting MYR in SLNs with Cou 6. The SD-Cou MPs exhibited a MMAD of approximately 2.5 µm, FPF exceeding 80%, and EF greater than 90%.

### Fate of labeled SLNs & SD-MPs following intravenous and pulmonary administration in mice

**Fig. 5:**
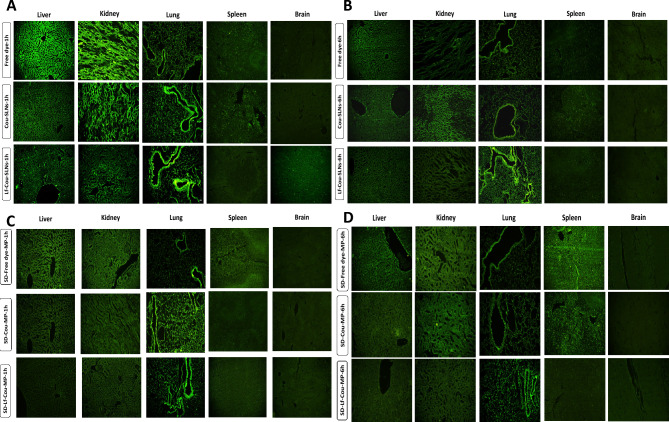
Fluorescence photomicrographs of tissues from different organs following the IV administration of Free dye solution, Cou-SLNs, and Lf-Cou-SLNs (**A**) 1 h; (**B**) 6 h. Fluorescence photomicrographs of tissues from different organs following inhalation of SD-Free dye MP, SD-Cou MP, and SD-Lf-Cou MP: (**C**) 1 h; (**D**) 6 h. (Magnification power 20X).

As proof of concept, the biodistribution of inhalable spray dried Cou-SLNs embedded microparticles *versus* nanoencapsulated Cou-6 and free coumarin dye was investigated. Further, the impact of lactoferrin targeting ligand in Lf-Cou-SLNs and Lf-Cou-SLNs embedded MPs was explored. The aforementioned formulations were tracked 1 and 6 h following local (inhalation) *vs* systemic (IV) administration. Figure 5 represents the biodistribution of fluorescent dye in various body organs including the lung, liver, spleen, kidney and brain by CLSM. After 1 h, IV-administered Cou-SLNs showed obvious accumulation in the airways and kidneys, whereas lactoferrin-targeted SLNs were preferentially accumulated in the respiratory tract, Fig. 5A, and remained distinct over 6 h, Fig. 5B. In comparison, the free dye was significantly distributed in the highly perfused organs (lungs, liver and kidney) one hour-post injection denoting high systemic exposure, Fig. 5A, and then rapidly cleared to other organs as noted by the faint fluorescence in the lungs after 6 h, Fig. 5B.

In contrast, the inhaled SLN-embedded microparticles (SD-Cou MP and SD-Lf-Cou MP) demonstrated preferential localization within bronchial and alveolar regions (Fig. 5C). This behavior can be related to soy lecithin phospholipids in the SLN formulation, that mimic pulmonary phospholipid surfactants and may act as a reservoir for the inhaled SLNs^35^. Of interest, SD-Lf-Cou MPs solely granted prolonged retention in the pulmonary tissues along 6 h highlighting the indispensable targetability and retention of lactoferrin, Fig. 5B,D. Active targeting via lactoferrin positioned the dye in the vicinity of alveolar cells, reduced systemic exposure and in turn minimized toxic side effects.

Overall negligible fluorescence could be noticed in brain tissues evidently related to the blood brain barrier.

Improved pulmonary deposition and retention via targeted SLNs-embedded MPs was consistent with previous reports^35–37^.

Fluorescent photomicrographs were analyzed using *Image J* software by quantifying the mean fluorescence intensity to evaluate pulmonary *vs* IV administration.

**Fig. 6:**
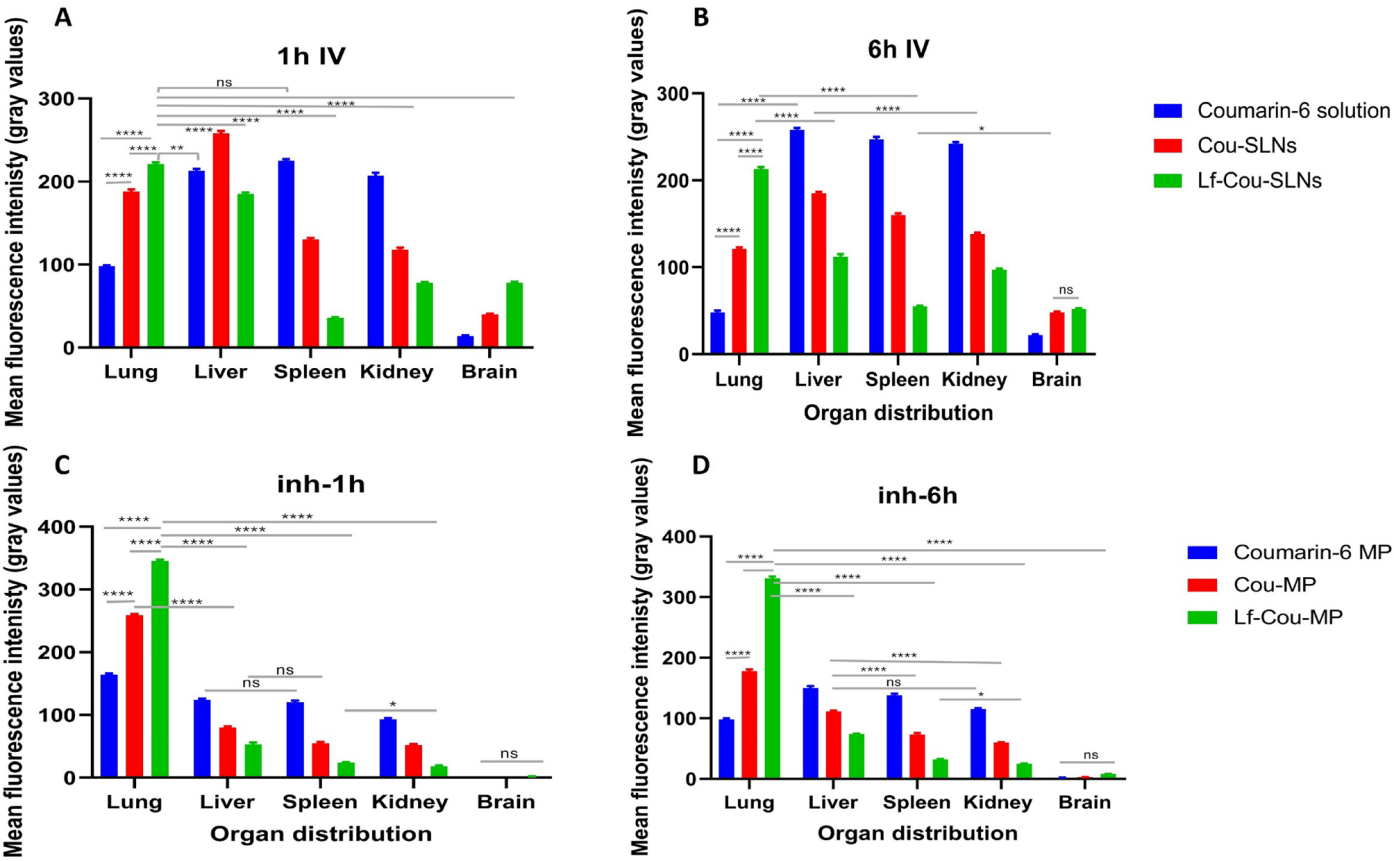
Fluorescent intensity of free dye, Cou-SLNs and Lf-Cou-SLNs detected in the tissue organs (**A**) 1 h- and (**B**) 6 h-post IV administration; Fluorescent intensity of SD-free dye MP, SD-Cou MP and SD-Lf-Cou MP detected in the tissue organs (**C**) 1 h- and (**D**) 6 h-post pulmonary administration.

IV-administered coumarin showed non-selective systemic distribution across the liver, spleen and kidneys (gray values > 200), Fig. 6A,while significantly weaker fluorescence was observed in the lungs and brain (98 and 14 Gy values, respectively; two-way ANOVA, Tukey’s multiple comparison test, *p* < 0.0001). In comparison, Cou-SLNs showed double the fluorescence intensity in the airways (188 Gy values) 1 h after IV injection (statistically significant increase *p* < 0.0001). Interestingly, extensive pulmonary deposition of lactoferrin-targeted SLNs was confirmed by fluorescence intensity over 220 Gy values, two-way ANOVA, Tukey’s Multiple comparison test (p < 0.0001). However, 6-h post IV revealed high pulmonary clearance rate of free coumarin and Cou-SLNs from the respiratory tract, as evidenced by fluorescence fading, while the fluorescence level was maintained > 210 Gy values along 6 h in case of IV-injected Lf-Cou-SLNs denoting targetability and prolonged pulmonary retention, Fig. 6B.

Comparatively, the inhalation of labelled SLN-embedded microparticles to mice provided 1.5- to twofold higher pulmonary deposition relative to the IV route, as confirmed by significant increase in fluorescence intensity 1 h post-administration of SD-Cou-MPs and SD-Lf-Cou-MPs to 259 and 345 Gy values, respectively (Fig. 6C; two-way ANOVA, Tukey’s multiple comparison test, *p* < 0.0001). Together with this localized pulmonary accumulation, inhaled microparticles provided significantly minor systemic exposure to the various body organs investigated (below 85 Gy values) equivalent to 1.7- to 2.3-fold lower fluorescence (*p* < 0.0001). Analysis of the confocal photomicrographs of the airways after 6 h revealed notable fluorescence fading while keeping the same pattern (Fig. 6D) that might be attributed to alveolar macrophage uptake^38^. According to former investigations in rats, clearance of ^3^H-labelled SLNs from the respiratory tract could be either by mucociliary escalator and/or biodegradation / erosion of the lipid-based nanocarriers^38,39^.

Current in vivo data demonstrated that inhalable Lf-targeted SLNs-embedded microparticles grant preferential pulmonary accumulation of the cargo as well as prolonged lung residence time. Moreover, the restricted off-target biodistribution and negligible systemic exposure give promises for higher safety and reduced adverse effects. In terms of cancer therapy, this would be considered a high impact achievement.

## Materials and methods

### Materials

Myricetin of high purity was acquired from Shanghai Tauto Biotech Co. Ltd in Shanghai, China. Gift samples of the lipids Gelucire 50/13 (G50/13) and Compritol-888-ATO (Cp) were received from Gattefossé, Lyon, France. Soybean phosphatidylcholine (Lipoid® S 100) with a purity above 96% was bought from Lipoid GmbH in Ludwigshafen, Germany. Coumarin-6 (Cou 6) was obtained from Polysciences Europe GmbH in Hirschberg, Germany. The blue, fluorescent stain Hoechst 33,342, which targets DNA in the nucleus of eukaryotic cells, was purchased from Thermo Fisher Scientific, USA. Sigma Aldrich (Schnelldorf, Germany) generously provided lactoferrin. Methanol and Ortho-phosphoric acid (HPLC grade) were obtained from Merk, Massachusetts, USA. A549 lung epithelial cancer cell line, Dulbecco’s modified eagle medium (DMEM), and fetal bovine serum (FBS) were obtained from the American Type Culture Collection (ATCC) in the USA and Gibco in Basel, Switzerland. The sodium sulfite was obtained from El-Nasr Pharmaceutical Company in Cairo, Egypt.

### Methods

#### Formulations of the lipid-based delivery systems

##### Preparation of MYR-CPX-SLNs ± Lf

To investigate the antitumor activity, SLNs loaded with MYR-PH-CPX were prepared as previously detailed by Nafee et al.^5^. Briefly, MYR and Lipoid® S 100 in molar ration (1:4) were dissolved in a mixture of ethanol and acetone (1:1). The solvent was then evaporated to create MYR-PH-CPX. The latter was introduced into the molten lipid mixture (G50/13 and Cp) at 55 °C, and then hot water was gradually added under magnetic stirring (600 rpm for 10 min) followed by homogenization using high shear homogenizer T18 ULTRA-TURRAX® (IKA, Staufen im Breisgau, Germany) at 8000 rpm for 15 min and probe sonication (Julabo Sonicator, model USR-3, Germany) for 10 min at 55 °C. The nanoemulsion was ultimately stirred at room temperature to obtain solidified (MYR-CPX-SLNs).

Lf-coupled SLNs (referred to as Lf-MYR-CPX-SLNs) were obtained by adding aqueous solution of Lf (1% w/v) as a targeted ligand to the SLNs while stirring. Lf concentration was selected following preliminary trials using 10 Lf concentrations ranging from 0.2 to 6% w/v (Supplementary materials, Table S1).

##### Preparation of coumarin-labeled SLNs ± lactoferrin

Coumarin-6 was incorporated into SLNs for visualization purposes to verify the in vitro cellular uptake and in vivo biodistribution of labeled SLNs via confocal laser scanning microscopy (CLSM) and fluorescence microscopy, respectively. Cou-SLNs and Lf-Cou-SLNs were prepared as described above by adding coumarin-6 (10 µg/ml) instead of MYR.

##### Preparation of lyophilized SLNs-embedded microparticles (SD-MPs)

MYR-CPX-SLNs were spray dried in presence of a carbohydrate carrier comprising maltodextrin, leucine and mannitol at a constant carrier to SLNs weight ratio (3:1). The optimum composition of the carrier was selected based on preliminary experiments as displayed in Table 5.

**Table 5:** Composition of spray-dried microparticles.

Formula code	Composition of MPs (w:w) ratio				
	MYR SLNs	Cou-SLNs	Mannitol	Maltodextrin	L-leucine
SD-MYR-CPX-SLNs	1	–	–	–	–
SD-MP1	1	–	3	–	–
SD-MP2	1	–	2	–	1
SD-MP3	1	–	1.5	0.75	0.75
SD-MP4	1	–	0.75	1.5	0.75
SD-Cou-MP	–	1	1.5	0.75	0.75

For in vivo inhalation studies, SD-Cou MP and SD-Lf-Cou MP were prepared by spray drying Cou-SLNs and Lf-Cou-SLNs, respectively.

Spray drying was conducted using the Spray Dryer B-90 (Büchi AG, Flawil, Switzerland) using the following experimental parameters (input temperature 110 °C, output temperature 55 °C, aspiration rate 100%, pump rate 15%, air flow rate 320 L/h). Collected SD-MPs were stored protected from moisture at room temperature for future investigations.

#### Characterization of the delivery systems

##### Colloidal properties and morphology of SLNs

The average particle size, size distribution (PDI) and zeta potential of SLNs prediluted with MilliQ water were determined using the Malvern Zetasizer-Nano (Malvern, UK). Data were represented as the average of triplicate measurements ± standard deviation. SLNs morphology was examined by transmission electron microscopy (TEM, JEM-100CX, JEOL, Japan), following negative staining with uranyl acetate.

##### Qualitative and quantitative confirmation of lactoferrin coupling to SLNs

IR spectra of Lf, specific MYR-CPX-SLNs, and Lf-MYR-CPX-SLNs were obtained by Fourier Transform Infrared FTIR spectroscopy (Perkin Elmer, USA) to confirm that Lf was coupled to the surface of SLNs.

Quantitatively, Lf concentration bound to Lf-MYR-CPX-SLNs was determined indirectly by Bradford assay. Briefly, SLNs (Lf-F6, theoretical Lf content 1% w/v, Table S1) were purified by centrifugal ultrafiltration using Centrisart-I^®^, MWCO 100 KDa (Sartorius Lab Ltd., Stonehouse, UK). Unbound Lf was quantified in the supernatant by mixing equal volume of the supernatant with Coomassie blue G dye solution (10% w/v), followed by 200-time dilution with purified water prior to absorbance measurement at 595 nm against a blank of dye solution. The total concentration of Lf in 1 ml SLNs was determined as described above without purification. Plain SLNs were evaluated in parallel as a control. Lf coupling efficiency was calculated using the following equation:

$${\% Lf coupling efficiency }= \frac{Total conc. of Lf in 1 ml SLNs-Conc. of unbound Lf in supernatent}{Total conc. of Lf in 1 ml SLNs} \times 100$$

##### Evaluation of the entrapment efficiency (EE) and in vitro release of MYR

To determine the encapsulation efficiency, Lf-coupled/uncoupled MYR-SLNs were purified using Centrisart-I^®^, MWCO 20 kDa (Sartorius Lab Ltd., Stonehouse, UK). The entrapped MYR was extracted with methanol and quantified using HPLC. In brief, samples (n = 3) were injected into a reversed-phase system with a Zorbax Eclipse XDB-C18 column. The elution was carried out at a flow rate of 1 ml/min using isocratic elution with 80% methanol (A) and 20% O-phosphoric acid solution 0.2% at pH 3.5 (B). The chromatograms were detected at λ 378 nm with a retention time (RT) of 2.5 min and a total run duration of 3.6 min. HPLC analysis was described in details in the Supplementary materials as validated earlier^11^.

MYR release experiment was conducted by dialysis in 50 ml hydroethanolic dissolution medium (1:1 v/v), in a shaking water bath at 100 strokes/min, at 37 ± 0.5 ℃. At predetermined intervals, samples were quantified using the aforementioned HPLC method to determine the %MYR released.

##### Microparticles characterization

Spray dried microparticles (SD-MP1 to SD-MP4) were characterized in terms of mean diameter, particle morphology, yield and percentage of drug recovery.

The particle size of spray-dried microparticles was determined by laser diffraction analyser using isopropyl alcohol as a suspending medium. Microparticle morphology was examined by scanning electron microscopy (SEM). To determine the drug content, the appropriate weights of microparticles were dissolved in methanol/water mixture. MYR in the filtrate was quantified by HPLC as described above. Particle yield represents the percentage of the mass microparticles obtained per total mass of solids introduced.

The flow properties were evaluated by determining the angle of repose, Carr’s index (CI), and Hausner ratio (HR) as described elsewhere^5^.

Drug-excipients incompatibility was verified by differential scanning calorimetry (DSC) and FTIR Spectroscopy of the drug and excipients individually as well as microparticles (SD-MP2 and SD-MP3).

Aerosol deposition was assessed as previously reported^5^. Briefly, a capsule containing SD-MP (20 mg) was instilled in the Aerolizer® inhaler device coupled to Andresen Cascade Impactor (ACI). The capsule was punctured to enable the powder to flow through the impactor at a flow rate of 28.3 L/min. MYR was extracted from microparticles collected from the throat and impactor stages (Stages 0–7) with methanol. The drug concentration in each stage was determined by HPLC as described above. The following aerosolization parameters: mass median aerodynamic diameter (MMAD), emitted dose (ED), emitted dose fraction (EF), fine particle fraction (FPF) and geometrical standard deviation (GSD) were determined as previously reported^5^.

#### In vitro assessment on pulmonary cell culture model

##### Antitumor activity of targeted and untargeted MYR-CPX-SLNs

The anticancer activity was investigated on A549 lung carcinoma cells by MTT assay. Detailed assay procedure is described in the Supplementary materials. In Corning 96-well plates, A 549 cells (5 X 10^4^ cells per well) were incubated with either MYR-PH-CPX, MYR-CPX-SLNs or Lf-MYR-CPX-SLNs equivalent to MYR concentrations from 10–200 µM for 24 h. After washing, A549 cells were incubated with MTT for 4 h. Formazan crystals were dissolved with DMSO. Absorbance was measured spectrophotometrically at 570 nm using microplate reader (SunRise, TECAN Inc, USA). The % cell viability was calculated relative to positive control cells treated with culture medium and negative control representing dead cells treated with Triton X. The experiment was carried out in triplicate. The IC_50_, denoting the dosage required to induce 50% cell death, was determined by analyzing the dose–response curve using GraphPad Prism software (San Diego, CA, USA).

##### Cellular uptake of Cou-SLNs in A549 cells

The uptake efficiency of the labelled SLNs (Cou-SLNs and Lf-Cou-SLNs) in A549 cells was compared to free coumarin dye. Concisely, A549 cells (12,500 cells/ml) placed on 8-chamber slides (IbiTreat®, ibidi GmbH, Graefelfing, Germany) were incubated for 4 and 24 h with the aforementioned SLNs at concentration equivalent to 6 X 10^6^ mg Cou 6. Untreated cells were examined as control. The nuclei of the fixed cells were stained blue with Höchst 33,342 and then examined by CLSM (Leica Microsystems, Wetzlar, Germany) at λ_ex_ 450 nm, λ_em_ 505 nm. Z-stacks (40 stacks, 14 µm) were captured by 3D-time laps imaging to distinguish internalized from membrane-bound particles.

Confocal images were processed with Leica AF software to determine the fluorescence intensity per unit area.

#### Pharmacodynamics of SLNs following in vivo inhalation *vs* IV administration

##### Experimental animals and study protocol

The biodistribution study was conducted in mice to compare the pharmacodynamics of Lf-targeted *versus* untargeted SLNs following both systemic (IV) and local (inhalation) administration.

The study was conducted on male Swiss albino mice, aged 4–5 weeks, 15–20 g, maintained at 25 ℃ and 50% RH. Mice were divided into six groups (four mice each) as depicted in Table 6. They were then fasted overnight, with no restrictions to water access before treatment.

**Table 6:** Animal groups and study protocol for biodistribution experiment.

Gp No	Sample	Administration route
Gp I	Free Cou	IV
Gp II	Cou-SLNs	IV
Gp III	Lf-Cou-SLNs	IV
Gp IV	SD-Cou-MPs	Inhalation (5 mg)
Gp V	SD-Cou-SLNs-MPs	Inhalation (5 mg)
Gp VI	SD-Lf-Cou-MPs	Inhalation (6 mg)

All experimental procedures were conducted in full accordance with the ARRIVE guidelines and adhered to the European Parliament Directive 2010/63/EU concerning the use of animals in scientific research. The study protocol received ethical approval from the Animal Care and Use Committee of the Faculty of Pharmacy, Alexandria University, Alexandria, Egypt (Approval ID: ACUC17/16).

The IV injection was administered in normal saline through the tail vein while, a DP-4 M insufflator (Penn-Century Inc., Philadelphia, PA, USA) was applied pulmonary delivery of microparticles as detailed by Roa et al.^40^. The dose of the fluorescent dye was equivalent to to 313 ng Cou-6 in all samples. Group V and VI received inhaled MP (5 and 6 mg, respectively, containing 1.25 mg Cou-SLNs and 1.5 mg Lf-Cou-SLNs, respectively.

##### Biodistribution study

Two mice per group were euthanized 1- and 6-h post administration and fixed after coronary heart perfusion. Subsequently, the lungs, liver, spleen, kidney, and brain were gathered and preserved in a solution of 10% neutral formalin.

Following fixation, tissues from each organ (2–3 mm thick) were trimmed then embedded in paraffin block. Sections (2 μm thick cut using a microtome) were mounted on microscope slides, using normal histological protocols.

##### Fluorescence microscopy

Tissue sections (n = 4) were examined using a fluorescence microscope (Olympus BX 41, Olympus America Inc., Melville, NY, USA) equipped with Plan Achromat N 20X and 40X Objective Lenses. The green-labelled SLNs were analyzed using the Image J software.

#### Statistical analysis

Data was presented as the mean ± standard deviation and analyzed using GraphPad Prism 0.8 (GraphPad Software Inc., USA). One- and two-way ANOVA were performed to determine statistical significance.

## Conclusions

Specificity, efficiency and safety are concrete milestones in cancer therapy. In this regard, the flavonoid myricetin, a potential anticancer nutraceutical, was complexed with soybean phosphatidylcholine and subsequently encapsulated in surfactant-free solid lipid nanoparticles. Site-specific delivery was endeavored via surface functionalization with lactoferrin ligand as well as formulation of nano-embedded respirable microparticles. This approach ensured promoted anti-tumor activity and significant uptake in A549 cells. The inhaled microparticles exhibited an average MMAD of 2.77 µm and FPF of 81.23%, confirming their suitability for deep airways deposition.

In vivo studies in rats revealed a 1.5- to twofold greater accumulation of inhaled fluorescently labeled microparticles in the lungs compared to the intravenous route, along with prolonged pulmonary retention over 6 h and minimal distribution to off-target organs such as the liver, kidneys, spleen, and brain. Data give promises for non-invasive, localized, safe and efficient cancer therapy.

## Supplementary Information

Supplementary Information.

## Data Availability

-Authors ensure that data are preserved following best practices and are retrievable for reanalysis. Data also comply with transparency and reproducibility standards of Scientific Reports. The datasets generated and/or analyzed during the current study accurately reflect the original and are available on reasonable request.
