# Supplementary material for: Inhalable herbal nanotherapeutics targeting lung carcinoma
Source: Sci Rep. 2026 Feb 2;16:4530. doi: 10.1038/s41598-025-18460-6 (PMC12868786; doi:10.1038/s41598-025-18460-6)
Supplement: Supplementary file 1 — Supplementary Information. [file 41598_2025_18460_MOESM1_ESM.docx]

**Inhalable herbal nanotherapeutics targeting lung carcinoma**

**Dina M. Gaber ^a^, Noha Nafee ^b,c,*^ , Maged W. Helmy** ^d^**, and Osama Y. Abdallah^b^**

*^a^ Department of Pharmaceutics, Division of Pharmaceutical Sciences, College of Pharmacy, Arab Academy for science, Technology and Maritime Transport, Alexandria 1029, Egypt*

*^b^ Department of Pharmaceutics, Faculty of Pharmacy, Alexandria University, Alexandria 21521, Egypt (Permanent address)*

*^c^ Department of Pharmaceutics, College of Pharmacy, Kuwait University, 13110 Kuwait (Present address)*

*^d^ Department of Pharmacology, Faculty of Pharmacy, Damanhour University, Damanhour, Beheira 22516, Egypt*

**Supplementary materials**

***Corresponding authors:**

***Prof. Dr. Noha Nafee**

**Current affiliation:**

Department of Pharmaceutics, College of Pharmacy, Kuwait University, P.O. Box 24923, Safat

13110 KUWAIT

Phone: +96524636072 Fax: +96524636843

Email: [noha.nafee@ku.edu.kw](mailto:noha.nafee@ku.edu.kw)

***Dr. Dina Gaber**

**Current affiliation:**

Department of Pharmaceutics, Division of Pharmaceutical Sciences, College of Pharmacy, Arab Academy for science, Technology and Maritime Transport, Alexandria 1029, Egypt

Phone: +201228442635

Email: [dinagaber@aast.edu](mailto:dinagaber@aast.edu)

**Article submitted to Scientific Reports**

**Supplementary materials**

**Table S1: Composition and characteristics of Lf-coupled and uncoupled MYR-CPX-SLNs**

| Formula code | Particle composition* | Mean SLN diameter (nm) ± SD | Polydispersity index (PDI) ± SD | Zeta potential (mV) ± SD |
| --- | --- | --- | --- | --- |
|  | **Ligand (Lf, % w/v)** |  |  |  |
| MYR-CPX-SLNs | ___ | **75.28** ± **0.738** | **0.27** ± **0.009** | **-26.5** ± **4.38** |
| Lf-F1 | 0.2 | **76.63** ± **1.049** | **0.304** ± **0.013** | **-17.7** ± **2.73** |
| Lf-F2 | 0.4 | **81.63** ± **7.523** | **0.314** ± **0.014** | **-9.4** ± **1.52** |
| Lf-F3 | 0.5 | **88.16** ± **1.467** | **0.328** ± **0.008** | **-8.6** ± **0.64** |
| Lf-F4 | 0.6 | **93.34** ± **0.472** | **0.337** ± **0.021** | **-7.5** ± **1.09** |
| Lf-F5 | 0.8 | **93.24** ± **0.63** | **0.349** ± **0.007** | **-4.59** ± **3.77** |
| Lf-F6** | 1 | **98.59** ± **0.474** | **0.355** ± **0.005** | **-3.81** ± **2.96** |
| Lf-F7 | 1.6 | **102.6** ± **0.848** | **0.42** ± **0.016** | **-3.31** ± **0.97** |
| Lf-F8 | 2 | **114.6** ± **5.97** | **0.461** ± **0.009** | **-1.51** ± **2.05** |
| Lf-F9 | 4 | **119.7** ± **1.69** | **0.478** ± **0.004** | **-1.08** ± **2.11** |
| Lf-F10 | 6 | **168.3** ± **0.754** | **0.541** ± **0.002** | **0.6** ± **0.66** |

**All formulations contain MYR (0.1% w/v) complexed with Lipoid S100 (1 %w/v); lipid mixture Gelucire 50/13 and Compritol (2.5 and 0.5 % w/v, respectively).*

***Lf-F6 is selected for further investigations.*

1. **HPLC method for quantification of MYR**

Standard MYR solution (10 mg% w/v) was prepared in methanol and stored at 4 °C in amber glass vial. The standard solution was diluted with methanol to prepare different concentrations (0.25–1.5 mg% w/v).

The HPLC analysis was performed using a system equipped with a reverse phase Zorbax Eclipse XDB-C18 column. The isocratic mobile phase, consisting of a mixture of methanol and 0.2% phosphoric acid solution pH 3.5 (80:20 v/v), was eluted at a flow rate 1 ml/min. The injection volume was 10 μl. The eluent was monitored by the diode array detector from 190 to 400 nm, and chromatograms were extracted at the wavelength of 378 nm. All determinations were performed at 25 °C. Triplicate injections were made for each concentration and chromatographed as under the previously described LC conditions. A calibration curve was constructed by plotting measured peak area versus corresponding MYR concentration and the regression line was fitted.

**Method validation**

**Linearity**

****The linearity of the proposed HPLC method was evaluated by analyzing a series of different concentrations for MYR. The linear regression equation was generated by least squares treatment of the calibration data. Under the optimized HPLC conditions, the measured peak areas at 378 nm were found proportional to concentrations, whereby; the standard calibration curve of peak area versus concentration was linearly correlated (R2= 0.9999) over the MYR concentrations from (0.125–1.5 mg% w/v).

**Detection and quantification limits**

The limit of detection (LOD) is defined as the concentration of the analyte which has a signal-to-noise ratio of 3:1. For the limit of quantification (LOQ), the ratio considered is 10:1. The LOD and LOQ values were calculated using the signal-to-noise ratio method and found to be 0.0067 and 0.0202 mg% w/v, respectively. Both LOD and LOQ values indicate that the proposed method showed low noise levels along with the high drugs responses which enable the quantitation and detection of low concentrations.

**Inter- and intra-day precision**

Both within-day (intra-day) and the between-day (inter-day) precision for the proposed method were studied by measuring 5 different concentrations for MYR using triplicate determinations for each concentration within the same day and next day. Results showed that the relative standard deviation for inter-day and intraday assay; RSD < 2%, percentage relative error (Er) =0.9679% proving the high repeatability and accuracy of the developed method for the estimation of concentration of MYR.

1. **Percentage of yield recovered and drug content**

The powder yield was simply calculated by dividing the weight of spray-dried powder (SD) by the total mass of solid introduced in the preparation submitted to spray drying as follows:

% Yield= $\frac{Mass of SD powder recovered}{Total mass of solid used in formulation}*100$ **…… Equation S.1**

1. **Particle size measurements**

The particle size of a powder formulation intended for inhalation is, together with the particle density, a prominent factor in the success of the formulation because it strongly influences the dispersion and sedimentation properties of the powder ^[1]^.

1. **Drug-Excipient Compatibility Study**
   1. **Differential Scanning Calorimetry (DSC)**

**
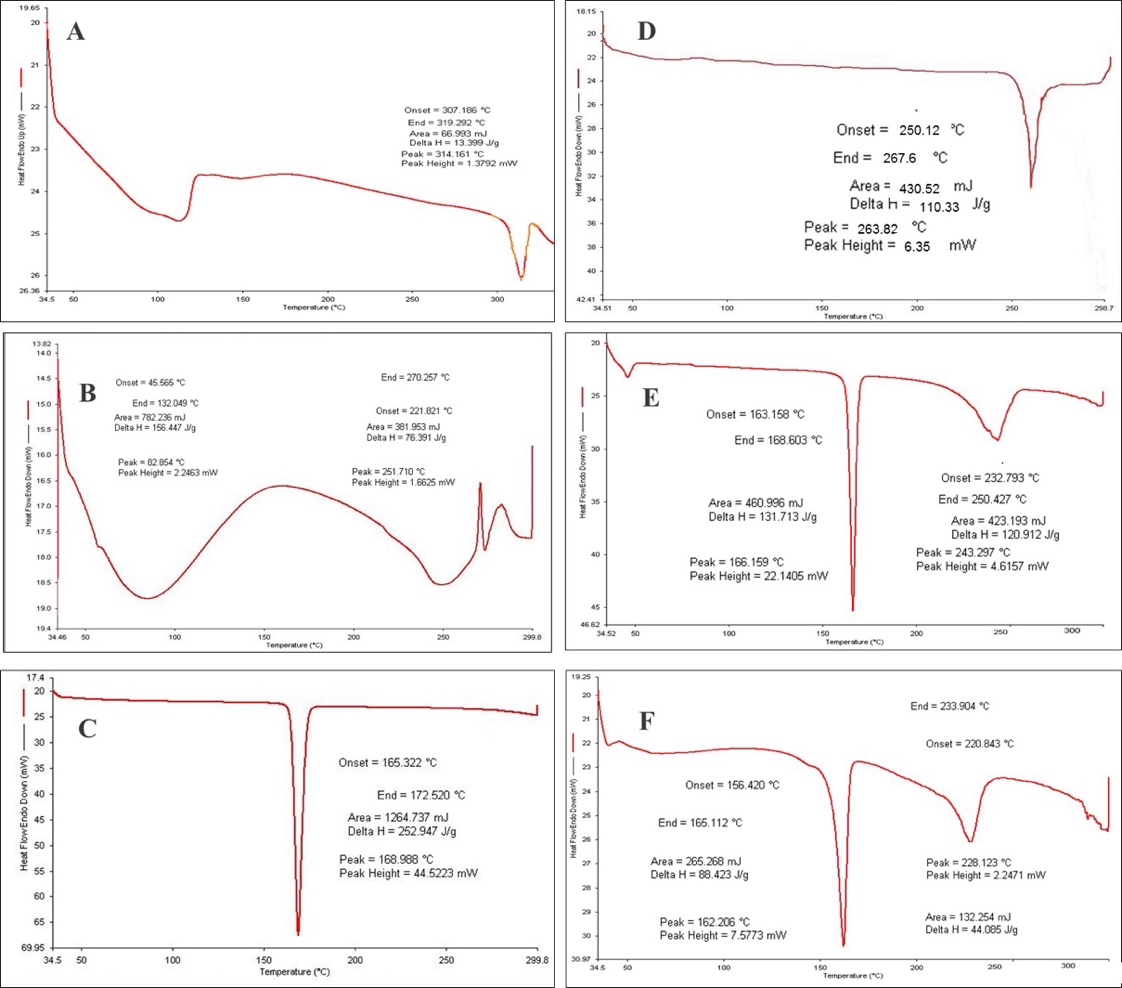
**

**Figure S.1:** DSC thermograms of spray dried MYR microspheres powder with its individual components; (A) Myricetin, (B) Maltodextrin, (C)Mannitol, (D) Leucine, (E) SD-MP2, and (F) SD-MP3.

### Fourier transform infrared (FTIR) spectroscopy

MYR showed IR absorption at wave number (cm^-1^) 3586.65-3282.31 (O-H stretching), 2942.33, 2855.4 (C-H stretching), 1660.5 (-C=O group), 1611.71-1517 (-C=C), 1462 (aromatic ring), 1377.06, 1325.97 (C-O-C). The IR spectrum of pure maltodextrin revealed the band at 3317.05 cm^-1^ due to O–H stretching, at 2925.027 cm^-1^ attributed to the sp3 C–H stretching, at 1647 cm^-1^ assigned to C-O stretching and 1419 cm^-1^ due to-CH_2_ bending. Further, pure maltodextrin spectrum presented peaks of 1147.38, 1076.89, and 928 cm^-1^. The peaks of 1147.38 and 1076.89 cm^-1^ are caused by stretching of the C-O bond and the 928 cm^-1^ peak due to the angular deformation of the CH and CH_2_ bonds, all from groups found in the carbohydrates ^[7]^. Mannitol showed a broad band at 3384-3277 cm^-1^ relating to O-H stretching vibration as well as a band at 2983.67 due to C-H stretching. The OH plane deformation of 1^ry^ and 2^ry^ alcohol was attributed to peaks 1280 and 1259 cm^-1^. The peaks at 1075 and 1017 are caused by 1^ry^ and 2^ry^ alcohol CO stretching. Furthermore, bands at 951, 926, 880 and 659 represented the fingerprint of mannitol ^[8]^. The aerosolization enhancer L-leucine molecules exhibited a characteristic peak at 2925 cm^-1^ due to its methyl group characterized by aliphatic CH_3_. At 1508 cm^-1^ assigned to the bending vibration of N-H band and at 1571.4 cm^-1^ attributed to the stretching mode of carbonyl group which is confirmed by the appearance of medium intensity band at 1405 cm^-1^ assigned to COO^-^ asymmetric vibration as previously reported by Ishak and Osman ^[4]^.


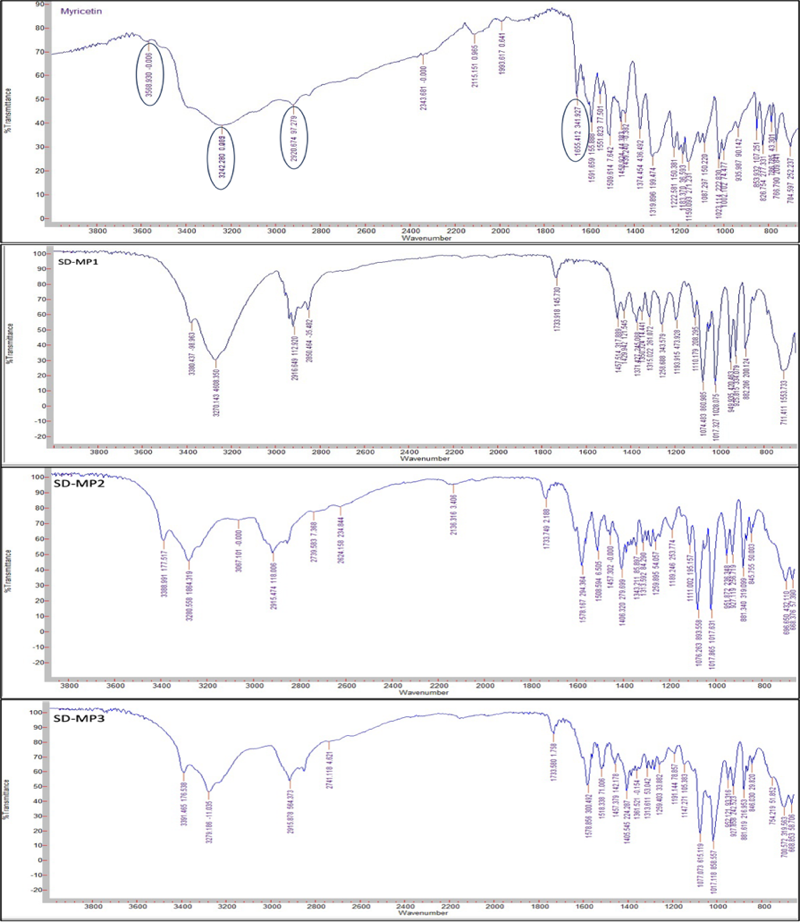


**Figure S.2:** FTIR spectra of SD-MPS powder (SD-MP1-SD-MP3) with its individual components and SD-MYR-CPX-SLNs**.**

1. ***In vitro* deposition**

The deposition pattern of different spray dried microparticles is illustrated in terms of amount deposited in µg per stage in Figures S.3. Generally, a minimum % deposited in the upper part of the respiratory tract would be favored.

**Figure S.3:** Drug distribution per discharge showing drug deposition at each stage for SD-MPs (SD-MP1-SD-MP4)

1. **MTT assay**

MTT assay is a colorimetric sensitive method to quantitatively assess cell viability/proliferation. The method is based on the reduction of the yellow tetrazolium salt MTT ( (3-[4,5-dimethylthiazol-2-yl]-2,5 diphenyl tetrazolium bromide)) to blue/violet formazan crystals by mitochondrial succinate dehydrogenase activity of viable cells. A linear relationship is established between the cell metabolic activity and absorbance.

The procedure recommended by the supplier was followed. After incubation of cell monolayer with different samples in the concentration range (10 – 200 µM) for 24 h. MTT reagent was added to washed cells and allowed to form formazan crystals for 4h. The crystals were dissolved with DMSO and the absorbance was measured at 570 nm.

Cell viability was determined as follows:

Where A (Test) is the absorbance obtained from the test sample, A (Negative control) is the absorbance obtained from dead cells treated with Trion X (zero viability) and A (Positive control) correspond to 100 % cell viability and represents the absorbance obtained from untreated cells (incubated with medium only).

Substance control (SLNs + assay reagents) showed no interference with the assay conditions.

**References**

1. Al-Qadi S, Grenha A, Carrión-Recio D, Seijo B, Remuñán-López C. Microencapsulated chitosan nanoparticles for pulmonary protein delivery: In vivo evaluation of insulin-loaded formulations. Journal of Controlled Release 2012;157(3):383–90.

2. Rockville M. The United States Pharmacopoeia 30, the National Formulary 25 US Pharmacopeial Convention. Electronic version 2007.

3. El-Gendy N, Berkland C. Combination chemotherapeutic dry powder aerosols via controlled nanoparticle agglomeration. Pharmaceutical Research 2009;26(7):1752–63.

4. Ishak RAH, Osman R. Lecithin/TPGS-based spray-dried self-microemulsifying drug delivery systems: In vitro pulmonary deposition and cytotoxicity. Int J Pharm 2015;485(1–2):249–60.

5. Elnaggar YSR, El-Massik MA, Abdallah OY, Ebian AER. Maltodextrin: a novel excipient used in sugar-based orally disintegrating tablets and phase transition process. AAPS PharmSciTech 2010;11(2):645–51.

6. Nafee N, Gaber DM, Elzoghby AO, Helmy MW, Abdallah OY. Promoted Antitumor Activity of Myricetin against Lung Carcinoma Via Nanoencapsulated Phospholipid Complex in Respirable Microparticles. Pharm Res 2020;37(82):1–24.

7. Krishnaiah D, Sarbatly R, Nithyanandam R. Microencapsulation of Morinda citrifolia L. extract by spray-drying. Chemical Engineering Research and Design 2012;90(5):622–32.

8. Bruni G, Berbenni V, Milanese C, Girella A, Cofrancesco P, Bellazzi G, et al. Physico-chemical characterization of anhydrous D-mannitol. Journal of Thermal Analysis and Calorimetry 2009;95(3):871–6.

9. Yu H, Teo J, Chew JW, Hadinoto K. Dry powder inhaler formulation of high-payload antibiotic nanoparticle complex intended for bronchiectasis therapy: Spray drying versus spray freeze drying preparation. International Journal of Pharmaceutics 2016;499(1):38–46.

10. Jacobs C, Müller RH. Production and characterization of a budesonide nanosuspension for pulmonary administration. Pharmaceutical Research 2002;19(2):189–94.

11. Malamatari M, Somavarapu S, Bloxham M, Buckton G. Nanoparticle agglomerates of indomethacin: The role of poloxamers and matrix former on their dissolution and aerosolisation efficiency. Int J Pharm 2015;495(1):516–26.

12. Sou T, Orlando L, McIntosh MP, Kaminskas LM, Morton DA V. Investigating the interactions of amino acid components on a mannitol-based spray-dried powder formulation for pulmonary delivery: a design of experiment approach. International Journal of Pharmaceutics 2011;421(2):220–9.

13. Kho K, Hadinoto K. Optimizing aerosolization efficiency of dry-powder aggregates of thermally-sensitive polymeric nanoparticles produced by spray-freeze-drying. Powder Technology 2011;214(1):169–76.

14. Osman R, Al Jamal KT, Kan PL, Awad G, Mortada N, Abd-Elhameed ES, et al. Inhalable DNase I microparticles engineered with biologically active excipients. Pulmonary Pharmacology & Therapeutics 2013;26(6):700–9.
